# Surprise!—Clarifying the link between insight and prediction error

**DOI:** 10.3758/s13423-024-02517-0

**Published:** 2024-05-14

**Authors:** Maxi Becker, Xinhao Wang, Roberto Cabeza

**Affiliations:** 1 Department of Psychology, Humboldt University Berlin, Berlin, Germany; 2 Center for Cognitive Neuroscience, Duke University, Durham, NC 27708 USA

**Keywords:** Insight, Prediction error, Aha experience, Surprise, Compound remote associates

## Abstract

The AHA experience, a moment of deep understanding during insightful problem-solving involving feelings of certainty, pleasure, and surprise, has captivated psychologists for more than a century. Recently, a new theoretical framework has proposed a link between the AHA experience and prediction error (PE), a popular concept in decision-making and reinforcement learning. This framework suggests that participants maintain a meta-cognitive prediction about the time it takes to solve a problem and the AHA experience arises when the problem is solved earlier than expected, resulting in a *meta-cognitive PE*. In our preregistered online study, we delved deeper into this idea, investigating whether prediction errors also pertain to participants’ predictions regarding the solvability of the problem itself, and which dimension of the AHA experience aligns with the meta-cognitive PE. Utilizing verbal insight problems, we found a positive association between the AHA experience and the meta-cognitive PE, specifically in regards to problem solvability. Specifically, the element of *surprise,* a critical AHA dimension, emerged as a key indicator of the meta-cognitive PE, while other dimensions—such as pleasure, certainty, and suddenness—showed no signs for similar relationships, with suddenness exhibiting a negative correlation with meta-cognitive PE. This new finding provides further evidence that aspects of the AHA experience, *surprise* in particular, correspond to a meta-cognitive PE. The finding also underscores the multifaceted nature of this phenomenon, linking insights with learning theories and enhancing our understanding of this intriguing phenomenon.

**Supplementary information:**

The online version contains supplementary material available at 10.3758/s13423-024-02517-0.

## Introduction

Many scientific discoveries and groundbreaking innovations have been the result of insights that have been described as thrilling moments of clarity and understanding. Those sudden understandings of a nonobvious problem involve connecting seemingly unrelated ideas or concepts and are usually accompanied by an “AHA!” experience (Danek et al., 2020; Dietrich & Kanso, 2010). People do not always experience insight or an AHA moment when they come up with new ideas or solve problems. But when they do, the idea or solution feels discontinuous, internally rewarding and surprising, including the subjective experience that it appeared suddenly and is certainly correct (Danek & Wiley, 2017; Kizilirmak et al., 2019; Metcalfe & Wiebe, 1987; Topolinski & Reber, 2010).

### AHA experience as (meta-cognitive) prediction error

What explains this fundamental difference in subjective phenomenology between insight and noninsight solutions? For more than a century, psychology has held an interest in understanding the essence of the AHA experience, leading to an extensive body of literature exploring both the behavioural and neurocognitive aspects of this phenomenon (for a review, see Becker et al., 2023), alongside theories about its phenomenology (Topolinski & Reber, 2010). For example, the AHA experience has been related to internal reward signals of having found the solution involving classical reward regions like the ventral striatum (Becker et al., 2023; Kizilirmak et al., 2016; Kizilirmak & Becker, 2023; Oh et al., 2020; Tik et al., 2018). However, this only explains the reward aspect, which is only one of the several dimensions of the AHA experience. In contrast, a new account attempts to connect the phenomenology of the AHA experience to the concept of *prediction error* (Becker & Cabeza, in press; Danek et al., 2015; Dubey et al., 2021; Friston et al., 2017; Savinova & Korovkin, 2022). This concept is widely known in decision-making and reinforcement learning (Sutton & Barto, 2018), due to its close conceptual proximity to surprise, reward and novelty. A prediction error (PE) generally describes a mismatch between a predicted outcome (i.e. prior experience derived from statistical regularities) and an actual outcome (i.e. sensory inputs or current thoughts; Rouhani et al., 2023). PEs may be classified into (1) perceptual/cognitive PEs, which refer to the size of the surprise of a perceptual/cognitive outcome, and (2) motivational PEs, which refer to the valence of the outcome—that is, whether an outcome is better or worse than expected (Den Ouden et al., 2012).

In the context of problem-solving, Dubey and colleagues (2021) argue that subjects are assumed to maintain a meta-cognitive model of their ability and prediction of when to solve a problem. Consequently, a PE arises when the solution is solved faster than expected, creating this sense of suddenness, surprise and internal reward (Dubey et al., 2021). As support for their assumptions, they conducted a large-scale online experiment, along with several simulation studies, where subjects were briefly presented with anagrams of varying difficulty for one second. They were then prompted to estimate how long it would take them to solve the anagram (ranging from 0 to 3 minutes). Subsequently, participants were asked to solve the anagrams and rate their AHA experience (scale of 1 to 7). The time PE was calculated by subtracting the actual solution time from the estimated solution time, which was then compared with the reported AHA experience. Their analysis revealed a significant positive correlation between participants’ time PE and their subjective AHA experiences (but note, the PE–AHA relationship may have been confounded by the varying difficulty of the anagrams).

Considering the AHA experience through the lens of a (meta-cognitive) PE is a promising approach not only because it has the potential to explain its distinct dimensions, such as pleasure and surprise, but also because it connects insight to a more general theory of (reinforcement) learning in psychology, potentially providing a more unifying account of this phenomenon (Dubey et al., 2021; Friston et al., 2017). When solving a problem via insight, the solution itself often seems to be completely unexpected (Kizilirmak et al., 2018; Metcalfe & Wiebe, 1987). Therefore, it is plausible that the AHA experience is associated not solely with a PE regarding the solution timing (Dubey et al., 2021), but also with several PEs concerning different aspects of the solution process, such as its general solvability or the content of the solution. What Dubey et al.’s (2021) study leaves further open is which aspects of the AHA experience (positive emotions, suddenness, certainty and surprise, amongst others; Danek & Wiley, 2017, 2020; Webb et al., 2016) best represent a meta-cognitive PE.

Savinova and Korovkin (2022) did not directly examine metacognitive PEs but explored the impact of solution expectancy on different dimensions of the AHA experience (pleasure, surprise, suddenness, and certainty) by manipulating subjects’ expectations across different problem sets. They compared a control group where solution approaches varied for each of the eight problems, with two experimental groups where the approach remained consistent except for the last problem. One experimental group had additionally similar problem structures. Results showed that as solutions became more expected (from problem 1–7 in experimental groups), surprise and (less consistently) pleasure decreased. Furthermore, in the experimental group with similar problem structures, pleasure and surprise additionally increased from the penultimate to the last problem. These results suggest a first link between solution expectancy and AHA experience, particularly with surprise and pleasure (no consistent relationship was found with suddenness and certainty). Yet, it remains unclear whether these particular dimensions of the AHA experience or others are linked to a metacognitive PE on a trial by trial level, as suggested by Dubey et al. (2021) and whether this relationship generalizes to other tasks.

### Current research and hypotheses

To further investigate those questions, we set up a pre-registered online study utilising verbal problems—compound remote associates (CRAs)—whose solution is often accompanied by an AHA experience (Bowden & Jung-Beeman, 2003). To estimate participants’ solution expectation, we first briefly presented them with the individual CRAs and asked them to evaluate the solvability of those problems (solution expectation). Subsequently, we had them solve the CRAs and rate their AHA experience. Importantly, similar to Savinova and Korovkin (2022), the AHA experience was divided into four different dimensions (internal *pleasure* of having found the solution and feeling that it appeared *suddenly*, *certainty* that the solution is correct and *surprise* about the solution result).

**Fig. 1.**
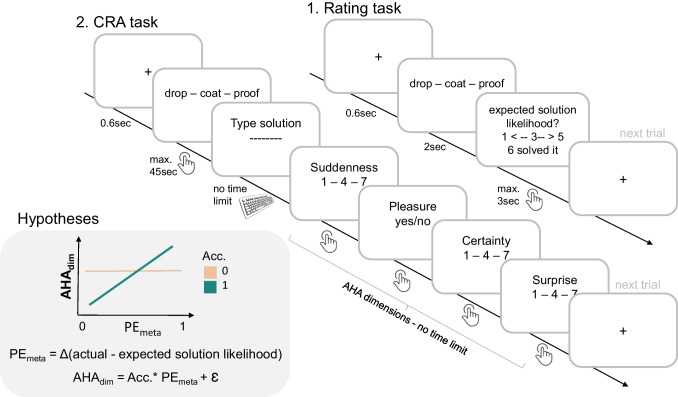
Experimental design and hypotheses. *Note.* CRA = Compound Remote Associates. AHA_dim_ = AHA experience dimensions; Acc. = Accuracy; Acc.0 = incorrectly solved trial; Acc.1 = correctly solved trial; PE_meta_ = meta-cognitive prediction error. Hypotheses: We expect a positive relationship between PE_meta_ and any of the AHA dimensions and this relationship should be modulated by accuracy. PE_meta_ is calculated as the difference between the actual solution and expected solution likelihood. The participant’s expected solution likelihood was measured via the Rating task. (Color figure online)

Under the assumption that some AHA experience dimensions represent a (meta-cognitive) PE of the problem’s solvability, we assumed that the difference between the solution expectation and the actual solution outcome should directly scale with the size of those AHA experience dimensions on a trial by trial basis. As we exclusively examine solved problems, where the solution outcome is inherently equal to or better than the expected outcome, we hypothesized a positive correlation between the meta-cognitive PE and the corresponding dimension of the AHA experience (see Fig. 1). Although not explicitly preregistered, we expect this positive correlation to be stronger in correctly solved trials, as only they are reliably interpreted as indicative of genuine insight.

## Methods

### Participants

The study was preregistered (https://aspredicted.org/ce7hu.pdf). Relying on the effect size from a study investigating the AHA experience as a prediction error (Dubey et al., 2021), we estimated a minimal sample size of *n* = 27 (ß = 95%, α = 5%). The study was conducted as an online format for an English-speaking population in MechanicalTurk, recruiting 45 participants. The local ethics committee of the Humboldt University Berlin approved the study. All participants received monetary compensation for their time on task. The only inclusion criterion was English-language proficiency because the task required high knowledge of English. For this, we adopted the Mill Hill vocabulary scale (Raven, 1960). Participants were excluded from further analyses if (1) they did not manage to choose the correct synonym for 5 out of 18 words from the Mill Hill vocabulary scale (*n* = 6), (2) showed no variance in their AHA rating (*n* = 0), or (3) in their solution likelihood rating (*n* = 0). Six participants were excluded from the study based on those criteria resulting in a final sample of *n* = 39 [age (in years): *M* = 43.2, *median* = 44; *SD* = 11.2; range: 27–65, 48.7% females.

### Materials and procedure

#### Materials

The stimulus material consisted of 60 normed Compound Remote Associates (CRA) published elsewhere (Bowden & Jung-Beeman, 2003). Those verbal tasks consist of three presented target words (e.g., *reading, service, stick*), and the goal is to find a solution word (*lip*) that can be appended in front or in the back of every one of the target words building a meaningful compound, respectively (*lip reading, lip service, lip stick*). Based on the norms, mean accuracy was 51.0% (*SD* = 24.4%; max = 97%; min = 10%) and solution time was 10.5 sec (*SD* = 3.49 sec, min = 4.12 sec; max = 18.69 sec). A list with all CRAs selected for this study in the experiment can be found in the Supplement (Table S1).

#### Procedure

The online experiment was programmed in Inquisit (Version 4.0; Inquisit, 2012), took approx. 45 minutes and was divided into four different tasks. To be eligible for participation in the experiment, participants first completed the Mill Hill task, followed by the execution of the Rating task, Word fluency task, and finally CRA task (explained in more detail as follows).

#### Rating task

After task instructions, participants first received nine practice trials to get used to the task. For the test trials, they received all 60 CRAs in a randomized order for 2 seconds and were subsequently asked about their solution expectation: “How likely do you think you can solve the problem on a scale between 1 (*very unlikely*) and 5 (*very likely*)?” After explaining the goal of the task to them, they were specifically instructed not to solve the individual items but to provide a personal or subjective estimate (“gut feeling”) of how likely they think they could solve the task. To enforce that participants do not overthink their respective estimate or try to solve the CRA, response time for every rating was limited to max 5 seconds (excluding the 2 seconds stimulus presentation). Average response time was 0.96 sec (*SD* = 0.74 sec). Trials where participants did not provide a rating within a 5-second time window were timed out and excluded from all further analyses (0.3% of all trials). Participants could also indicate whether they had already solved the trial within this 2-second time window; those trials were excluded from all further analyses (8.2% of all trials).

#### Word fluency task

In this short task, participants were asked to write down as many animals and plants as they can think of in one minute each. This short task was included primarily to distract the participants from thinking about the previously shown CRAs and their solutions as this might bias the results.

#### Compound Remote Associates (CRAs)

After a short task instruction including two practice trials, participants received the same 60 CRAs in a randomized order again that they had already seen in the Rating task. This time they were asked to solve them within max 45 seconds and if they failed to do so a new trial would start. They were instructed to press their solution button as soon as they found the answer and type in their respective solution word. Subsequently, they were asked to rate how they experienced their solution in relation to (1) *suddenness*, (2) *pleasure*, (3) *certainty,* and (4) *surprise* (see next paragraph) without a time limit. After providing all responses, a new trial would start.

### Insight assessment

Insight is typically assessed using self-ratings of the AHA experience, previously quantified as a binary variable denoting its presence or absence (Jung-Beeman et al., 2004; Kounios & Beeman, 2014). However, more recent investigations have revealed that the AHA experience actually constitutes a continuous phenomenon comprising several dimensions. These dimensions include (1) the extent of positive emotional response upon discovering the solution, (2) the perceived suddenness of the solution’s emergence, (3) the level of certainty regarding the correctness of the solution, and (4) the degree of surprise elicited by the solution, among other dimensions (Danek et al., 2014; Danek & Wiley, 2017, 2020; Webb et al., 2016). Therefore, we assessed insight via the AHA experience on a continuous scale and split it into those four main components (positive emotion/pleasure, certainty, suddenness, surprise). Note, however, there is still no consensus about which components make up the AHA experience, resulting in some researchers focussing more on the suddenness or emotional/pleasure component (Kounios & Beeman, 2014; Tik et al., 2018) and others on the surprise component (Gick & Lockhart, 1995) reducing comparability between studies. The different concepts were described to the participants as follows:

“Consequently, you are asked HOW you experienced finding the solution: Here, we ask about four different aspects of the AHA experience: suddenness, pleasure, certainty, & surprise that can, but don’t always, have to coincide.”

***Suddenness:*** “Did the solution come to you suddenly, or did you increasingly approach the solution in a stepwise manner? (scale: 1 [*stepwise*]–7 [*sudden solution*]).”

***Pleasure:*** “Did you experience a positive emotion (pleasure) upon finding the solution? yes/no.” 

***Certainty:*** “When the solution first appeared to you (before evaluation), how certain were you that the solution is correct? (scale 1–7).”

***Surprise:*** “How surprising does the solution result seem to you? (scale 1–7).”

Note, participants might be surprised not only by the moment of insight but also by the solution’s content. For example, they might not have expected that the solution, like “dog,” falls within the semantic category of mammals/animals when first given the task. To account for this, we allowed participants to interpret the nature of their surprise and simply referred to it as “surprise about the solution result” in the instruction and during the rating. In order to prevent participants from forgetting each dimension’s meaning, we also provided them with descriptions for each dimension during each individual rating. However, the possibility of idiosyncratic interpretations of these other dimensions cannot be entirely ruled out.

#### Measurement model for AHA experience

**Fig. 2:**
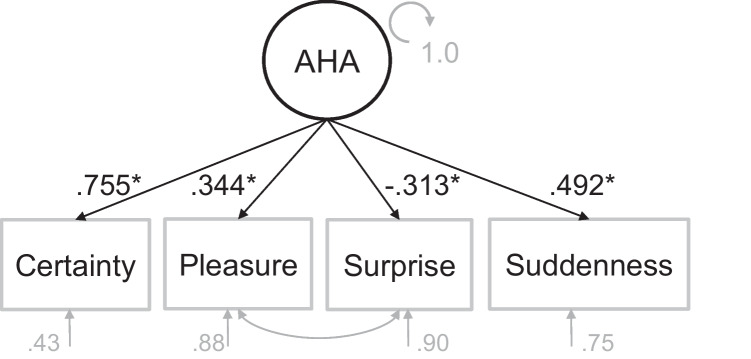
Measurement model: Latent AHA Experience factor loading onto four different AHA dimensions. *Note*. Asterisk indicates significant factor loading at *p* < .001

To demonstrate that those four dimensions form part of the AHA experience for the current data set, we calculated a measurement model for a latent AHA experience factor from those four dimensions (*suddenness, pleasure, certainty, surprise*) for correctly solved CRA items. The latent factor was estimated within a confirmatory factor analysis (CFA) in R using the lavaan package and its default settings (Version 0.6-15; Rosseel, 2012). In order to ensure that the relationships among the AHA dimensions in the measurement model remain unbiased by factors such as difficulty or individual differences between subjects or items, we utilized residualized data in the confirmatory factor analysis (CFA). That is to say, we first calculated a (general) linear mixed model for every AHA dimension controlling for solution time, trial number, as well as random subject and item effects (following this formula: AHA_dimensions_ ~ RT + trial# + (1|subject) + (1|item) + Ɛ). The resulting residuals were entered into the CFA. The measurement model was estimated via the robust maximum likelihood estimator and the resulting fit was evaluated via the exact chi-squared goodness-of-fit statistic as well as comparative fit indices such as Bentler’s comparative fit index (CFI), root-mean-square error of approximation (RMSEA), and standardized root-mean-square residual (SRMR). Accepted thresholds indicating good model fit are RMSEA ≤ .05, SRMR < 0.1, and CFI ≥ = .95 (Hu & Bentler, 1998, 1999; Schermelleh-Engel et al., 2014). To improve the model fit, we additionally specified covariances between the variables *pleasure* and *surprise* (see Fig. 2).

#### **Calculation of meta-cognitive prediction error (PE**_**meta**_**)**

We defined the PE_meta_ related to the solvability of a problem as the difference between the actual and expected solution outcome consistent with previous work (Den Ouden et al., 2012). The expected solution outcome was measured via the solution likelihood rating in the Rating task and the resulting values (1 = *very unlikely solvable* to 5 = *very likely solvable*) were normed between 0 and 1. The actual solution outcome (i.e., participants pressing the solution button under the belief they had found a solution regardless of its accuracy) was set to 1 (*very likely solvable*) as this relates to the highest possible solution likelihood and subtracted from the expected solution outcome (solution likelihood).

$${PE}_{meta}= {1 -"expected\;solution\;likelihood"}_{{\text{normed}}}$$

Note, in this problem-solving context, the actual solution outcome can only be better or as good as the expected solution outcome, but never worse, because we only consider solutions.

### Data analyses

For statistical analysis, three general linear mixed-effects models (Baayen et al., 2008) were applied predicting variance in s*uddenness, pleasure, certainty,* and *surprise* (dependent variable) with PE_*meta*_ (independent variable) on a trial-by-trial basis (see equations, below). We assumed a positive relationship between at least one of the AHA experience’s main dimensions and PE_*meta*_ if those components reflect internal errors in predicting the solution. Because insight refers to correctly solved trials (Salvi et al., 2016), we assumed that the positive relationship between PE_meta_ and the AHA dimensions should be more strongly pronounced for correctly than for incorrectly solved trials indicative of a genuine insight (see Fig. 1). Therefore, we additionally estimated an interaction between PE_meta_ and accuracy in predicting dimensions of the AHA experience. We further corrected for solution time to account for task difficulty and trial order to account for signs of fatigue or habituation. Subjects and items were modelled as random intercepts.

1. AHA_dimensions_ ~ Acc. + RT + trial# + (1|subject) + (1|item) + Ɛ
2. AHA_dimensions_ ~ PE_meta_ + Acc. + RT + trial# + (1|subject) + (1|item) + Ɛ
3. AHA_dimensions_ ~ PE_meta_ * Acc. + RT + trial# + (1|subject) + (1|item) + Ɛ

*Note*. AHA dimensions are *pleasure*, *suddenness*, *certainty,* and *surprise*. RT = solution time. PE_meta_ = meta-cognitive prediction error; acc.= accuracy.

Pop-out solutions (<2 sec) were excluded from all analyses as they do not count as insight solutions (Becker et al., 2021). Note however, as this resulted in an exclusion of 42.5% of all trials, we additionally repeated the analyses including trials that were solved in <2 sec. Importantly, the main results did not change significantly. Because pleasure was measured in a binary style, we modelled this variable via a binomial model (logit link function). All other models were modelled assuming a Gaussian link function. The *p* values were calculated via likelihood-ratio tests testing the baseline model (1) without PE_*meta*_ against the full model (2) with PE_*meta*_ and the full model (2) against the interaction model (PE_*meta*_ * accuracy) (3). For exploratory purposes, we additionally modelled a three-way interaction (PE_*meta*_ * accuracy * solution time) for *suddenness* to investigate whether solution time (i.e., task difficulty) modulated the unexpected negative relationship with PE_*meta.*_ Importantly, because we did not know which dimension of the AHA experience may relate to PE_*meta*_, all respective resulting *p* values were corrected for multiple comparison (Holm, 1979). Only the best model fit is being reported. All mixed-effects analyses were conducted in R (Version 4.2.0) using the glmmTMB package (Version 1.1.7; Brooks et al., 2017). The data as well as the analysis code have been made publicly available online (github.com/MaxiBecker/AHA_as_Prediction-Error).

## Results

On average, participants pressed the solution button in 85.3% (*SD* = 16.2%) of all CRAs and they solved 64.5% (*SD* = 16.9%) of all trials correctly. Median solution time for all trials was 7.63 sec (*SD* = 2.8 sec) and 6.3 sec (*SD* = 2.6 sec) for correctly solved trials. The CRA solutions were rated as *pleasing* in 57% (*SD* = 31%) of all cases and on a scale from 1 to 7 they were perceived as *certain* (*M* = 4.8; *SD* = .96), *sudden* (*M* = 4.62; *SD* = .95), and *surprising* (*M* = 3.08; *SD* = 1.08). Furthermore, participants were able to predict whether they would be able to correctly solve a CRA problem or not, χ^2^(1) = 5.46, *p* = .019; odds ratio = 1.20.

### Latent AHA experience factor loads onto AHA dimensions

The model converged normally after 26 iterations. The chi-squared goodness-of-fit statistic, χ^2^(1) = .629, *p* = .428, was not significant suggesting no significant difference between the measurement model and the data. Practical fit indices confirmed a good fit of the model to the data (CFI = 1.00; RSMEA = .000; SRMR =.007). The latent insight factor loaded significantly positively onto *Certainty* (*λ* = .755, *z* = 11.79; *p* < .001), *Suddenness* (*λ* = .492, *z* = 10.79; *p* < .001), and *Pleasure* (*λ* = .344, *z* = 6.75; *p* < .001), and significantly negatively onto *Surprise* (*λ* = −.313, *z* = −5.922; *p* < .001) suggesting that all four variables contribute significantly to the latent AHA experience factor (see Fig. 2). In sum, those results confirm that all four AHA dimensions explain relevant variance of a latent AHA experience factor.

### Relationship between meta-cognitive prediction error and AHA dimensions

#### Certainty

**Fig. 3:**
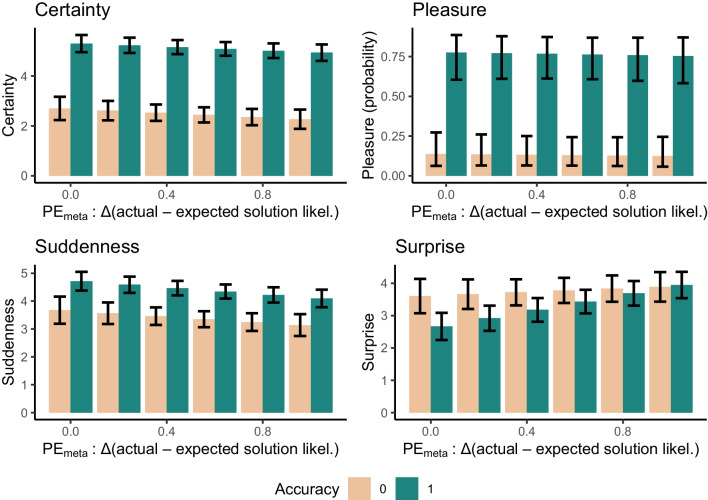
Meta-cognitive prediction error predicting AHA experience dimension *surprise. Note.* Values represent estimated marginal means. Error bars are 95% confidence intervals; likel. = likelihood; Accuracy 0 = incorrect solution; Accuracy 1 = correct solution

Accuracy, χ^2^(1) = 495.97, *p* < .001, *ß* = .57, CI [.53, .62], predicted the amount of certainty about the correctness of the solution. However, there was no evidence for PE_meta_, χ^2^(1) = 1.84, *p* = .17, ß = −.03, CI [−.07, .01], nor for an interaction between PE_meta_ and accuracy, χ^2^(1) = 0.35, *p* = .55 ß = .01, CI [−.03, .05], to predict variance in the amount of certainty about the solution (see Fig. 3, Table S2 in the Supplement).

#### Pleasure

Accuracy, χ^2^(1) = 276.85, *p* < .001, odds ratio = 24.60, CI [15.65, 38.68], predicted the amount of perceived pleasure upon finding the solution. However, there was no evidence for PE_meta_, χ^2^(1) = 1.88, *p* = .17, odds ratio = .70, CI [.42, 1.17], nor for an interaction between PE_meta_ and accuracy, χ^2^(1) = 1.38, *p* = .24, odds ratio = 2.0, CI [.63, 6.40], to predict variance in the amount of perceived pleasure (see Fig.3, Table S2 in the Supplement).

#### Suddenness

Both PE_meta_, χ^2^(1) = 6.52, *p*-Bonferroni = .043, ß = −.06 CI [−.11, .01], and accuracy, χ^2^(1) = 71.20, *p* < .001, ß = .23, CI [.18, .28], predicted the amount of perceived suddenness upon finding the solution (see Fig. 3). However, the relationship between PE_meta_ and suddenness was negative and therefore in the opposite than hypothesized direction. No evidence for a significant interaction between PE_meta_ and accuracy in predicting *suddenness* was observed, χ^2^(1) = 0.15, *p* = .69, ß = .01, CI [−.04, .06], hence, correctly and incorrectly solved trials contributed to this negative relationship (see Table S3 in the Supplement).

Given the unexpected negative relationship between *suddenness* and PE_meta_, we further explored whether this relationship is modulated by task difficulty (i.e., solution time). In fact, we found a three-way interaction between PE_meta_ * Accuracy * Solution Time, χ^2^(1) = 4.20, *p* = .04, ß = .05, CI [.00,.10]. A visual inspection of the interaction demonstrates that the negative relationship between PE_meta_ and *suddenness* for correctly solved trials is driven by quickly solved CRA items (~ 2.4 sec; see Fig. S1 in the Supplement).

#### Surprise

The amount of perceived surprise upon solution finding was significantly negatively predicted by accuracy, χ^2^ = 11.24, *p* < .001, ß = −.16 CI [−.21, −.11], and significantly positively predicted by PE_meta_, χ^2^(1) = 18.88, *p*-Bonferroni < .001, ß = .12, CI [.07, .27]. There was furthermore a trend for significance for a PE_meta_ * Accuracy interaction, χ^2^(1) = 3.68, *p* = .055, ß = −.12 CI [−.27, −.07]). A visual inspection of the interaction demonstrates that the positive relationship between PE_meta_ and *surprise* was mostly driven by correct solutions (see Fig. 3, Table S3 in the Supplement).

## Discussion

The AHA experience, an indicator of insight, is a complex construct with multiple dimensions, including pleasure, certainty, suddenness, and surprise about the solution. Recent suggestions propose a link between the AHA experience and meta-cognitive prediction errors (PE_meta_), reflecting the temporal difference between the expected and actual solution (Dubey et al., 2021). In this study, we further explored this link investigating whether PE_meta_ also relates to the expected solvability of the problem and which AHA dimension might reflect this aspect. We hypothesized a positive correlation between PE_meta_ and at least one AHA dimension, with a stronger effect for correct solutions, indicative of genuine insight (Danek & Salvi, 2020). As hypothesized, we found evidence that *surprise* was significantly predicted by PE_meta_, particularly for correct solutions. No other AHA dimension exhibited a significant relationship in the expected direction. Moreover, we observed a negative correlation between PE_meta_ and suddenness, contingent upon solution time and accuracy, as will be elaborated upon in the subsequent discussion.

### Relationships between different AHA dimensions and PE_meta_

The positive correlation between PE_meta_ and *surprise* is consistent with Savinova and Korovkin (2022) who found *surprise* to be most consistently related to solution expectancy. This relationship is further consistent with Dubey et al.’s, (2021) reinforcement learning account of insight suggesting that the AHA experience involves monitoring predictions about one’s interactions with the problem and “sudden insight surprises individuals about their own problem-solving ability,” leading to the AHA experience (Dubey et al., 2021, p. 14). This positive correlation further aligns with Friston et al.’s (2017) active inference framework by suggesting that the AHA moment represents a reduction in prediction error, as individuals update their beliefs about problem solvability. The positive correlation indicates that greater surprise during the AHA moment is linked to larger discrepancies between initial predictions and the actual solution, reflecting a significant revision of prior beliefs (Friston et al., 2017). Note, that albeit related, PE_meta_ and *surprise* are independent measures. PE_meta_ refers to the estimated *solvability* of the problem before the problem is solved, measured via the rating task. In contrast, *surprise* relates to the emotional evaluation of how unexpected the moment of solution or the solution *content* is perceived once the solution was found in the CRA task.

Finally, our results align with an fMRI study by Danek et al. (2015), demonstrating a connection between expectation violation in magic tricks and heightened activity in the anterior cingulate cortex. This brain region is frequently associated with prediction errors (Alexander & Brown, 2019) and commonly activated during insight (Becker et al., 2021; Dietrich & Kanso, 2010).

As assumed (albeit not explicitly preregistered), the assumed positive relationship between *surprise* and PE_meta_ was much more pronounced for correctly solved CRA items indicative of a genuine insight (see Fig. 3), although the interaction between accuracy and PE_meta_ only reached a trend for significance (*p* = .055). In contrast, *surprise* remained consistently high for inaccurately solved CRA problems as has been observed before (Danek & Wiley, 2017). This likely reflects the fact that for incorrectly solved problems, the solver was generally unable to make predictions about the solution content resulting in high surprise upon (incorrect) solution.

Our study found no evidence of a connection between PE_meta_ and the *pleasure* and *certainty* dimensions of AHA. While the null finding for the certainty dimension is consistent with the null finding for this dimension in Savinova and Korovkin’s (2022) study, they did observe a negative correlation between the level of solution expectation with (not only surprise but also) pleasure, although pleasure showed less consistent results. This also contrasts with Dubey et al.’s (2021) suggestion that the AHA experience is analog to a reward prediction error. However, their assessment treated the AHA experience as a composite measure, making it impossible to determine which AHA dimension drove the positive link with their PE_meta_ measure. Our null findings may stem from insufficient statistical power, due to significant interindividual differences related to trait reward sensitivity (Oh et al., 2020), despite our efforts to account for random subject effects in our analyses. Alternatively, PE_meta_ may be primarily associated with the *surprise* element of the AHA experience and less with *pleasure* and *certainty*. In contrast, *pleasure* and *certainty* may be more reflective of reward towards having found the solution irrespective of prior expectations (Kizilirmak & Becker, 2023; Oh et al., 2020) and how well the new solution fits into the solver’s existing knowledge base (Laukkonen et al., 2022). Consistently, we found that *pleasure* and *certainty* were influenced by accuracy, implying both dimensions were elicited from the discovery of the correct solution. This aligns with past research showing that *pleasure* and *certainty* are better predictors of accuracy than *surprise* (Webb et al., 2018).

Finally, PE_meta_ negatively predicted *suddenness*, indicating that participants who expected to solve the CRA problem were more likely to perceive the solution as sudden. While this finding may appear counterintuitive, it can be explained by considering the influence of task difficulty on both *suddenness* and expected solution likelihood, as we found an effect of task difficulty (here measured via solution time) on participants’ expected solution likelihood and *suddenness* ratings. According to spreading activation accounts of insightful problem solving (Becker et al., 2022; Bowers et al., 1990), simple CRA problems possess strong cue associations (e.g., drop, forest, cape) with the solution word (rain), leading to automatic preactivation of the solution upon cue presentation, thereby enhancing the sense of solvability (increased expected solution likelihood). At the same time, a solution may be perceived as *sudden* when the solution word was automatically activated including less controlled search processes indicative of simple problems (Becker et al., 2022). This is consistent with our finding that the negative relationship for *suddenness* and predictions of problem solving ability was mainly driven by simple problems solved in less than 4 seconds whereas the relationship ceases for (more difficult) problems solved later than that (see Fig. S1 in the Supplements).

Note, all four AHA dimensions were related to task difficulty but not in the same direction. While *suddenness*, *certainty* and *pleasure* were particularly high for easy problems, *surprise* increased with task difficulty, which probably explains the opposite factor loading on the latent AHA variable. Hence, to avoid a possible confound with task difficulty and understand the AHA experience’s diverse functions, it is important to assess its different dimensions, particularly *surprise*. In sum, all results combined suggest that the AHA experience is a multifaceted complex construct that reflects various cognitive functions, with *surprise* being positively associated with a PE_meta_ as previously suggested (Dubey et al., 2021). However, our results also suggest that PE_meta_ is likely not the only factor driving the AHA experience.

### Open questions

Numerous questions remain unanswered, presenting future avenues for exploration. For example, PE_meta_ has been observed to encompass various elements of the solution process, such as estimates regarding the timing of solution derivation (Dubey et al., 2021), as well as the general solvability of the solution (current study). To what extent do participants engage in meta-cognitive predictions concerning other aspects of the solution process depending on the type of problem, and how do these predictions interconnect with different AHA dimensions? Furthermore, certain dimensions of the AHA experience, such as *relief, drive to act*, and *impasse*, were not investigated in this study (Danek & Wiley, 2017; Webb et al., 2016). This begs the question of whether any of these dimensions might bear associations with PE_meta_. Moreover, participants likely lack awareness of all their various predictions concerning the solution process. Therefore, assessing implicit solution expectations of subjects (ideally integrating objective measures via ERP studies to quantify surprise) and comparing them with the AHA experience could enhance our understanding of how prediction errors manifest in the surprise dimension of the AHA experience. Further research is warranted to address these open questions.

### Conclusion

In current insight research, efforts are underway to make this phenomenon more compatible with existing learning theories by associating the AHA experience with (meta-cognitive) PEs commonly known in reinforcement learning (Dubey et al., 2021; Friston et al., 2017). This is an important step towards a more comprehensive explanation of the insight phenomenon. The present study fills an important gap in this endeavour and again stresses the fact that the AHA experience is a complex and heterogeneous subjective phenomenon signalling different cognitive functions about the phenomenon (Danek & Wiley, 2017; Webb et al., 2016).

## Supplementary information

Below is the link to the electronic supplementary material.

Supplementary file1 (DOCX 180 KB)

## Data Availability

The data for both experiments are available online (github.com/MaxiBecker/AHA_as_Prediction-Error), and the main experiment was preregistered (https://aspredicted.org/ce7hu.pdf).
