# Supplementary material for: Surprise!—Clarifying the link between insight and prediction error
Source: Psychon Bull Rev. 2024 May 14;31(6):2714–23. doi: 10.3758/s13423-024-02517-0 (PMC11680657; doi:10.3758/s13423-024-02517-0)
Supplement: Supplementary file 1 — Supplementary file1 (DOCX 180 KB) [file 13423_2024_2517_MOESM1_ESM.docx]

# Supplementary Material

| **Table S1** | | | |
| --- | --- | --- | --- |
| Stimulus material for experiment | | | |
| **CRAs** | **Solution** | **Acc in %** | **RT** |
| reading - service - stick | lip | 10 | 9.57 |
| cry - front - ship | battle | 18 | 13.69 |
| office - mail - hat | box | 21 | 17.23 |
| baby - spring - cap | shower | 28 | 7.58 |
| lift - card - mask | face | 33 | 12.79 |
| hold - print - stool | foot | 41 | 8.62 |
| light - birthday - stick | candle | 46 | 9.74 |
| piece - mind - dating | game | 46 | 15.34 |
| dress - dial - flower | sun | 51 | 7.78 |
| horse - human - drag | race | 56 | 12.14 |
| peach - arm - tar | pit | 67 | 10.01 |
| basket - eight - snow | ball | 72 | 10.87 |
| sleeping - bean - trash | bag | 82 | 6.8 |
| nuclear - feud - album | family | 85 | 9.48 |
| cane - daddy - plum | sugar | 97 | 5.45 |
| way - ground - weather | fair | 10 | 17.04 |
| stick - maker - point | match | 21 | 12.19 |
| note - chain - master | key | 26 | 12.68 |
| break - bean - cake | coffee | 33 | 14.04 |
| age - mile - sand | stone | 44 | 16.61 |
| foul - ground - mate | play | 46 | 9.33 |
| fly - clip - wall | paper | 49 | 11.02 |
| palm - shoe - house | tree | 51 | 13.9 |
| boot - summer - ground | camp | 54 | 4.46 |
| opera - hand - dish | soap | 62 | 7.92 |
| sage - paint - hair | brush | 69 | 9.88 |
| fish - mine - rush | gold | 74 | 9.07 |
| sandwich - house - golf | club | 82 | 9.1 |
| worm - shelf - end | book | 85 | 6.76 |
| night - wrist - stop | watch | 97 | 6.27 |
| wise - work - tower | clock | 13 | 13.32 |
| roll - bean - fish | jelly | 26 | 13.24 |
| force - line - mail | air | 28 | 13.9 |
| tail - water - flood | gate | 36 | 10.23 |
| change - circuit - cake | short | 41 | 10.07 |
| oil - bar - tuna | salad | 41 | 17.05 |
| tomato - bomb - picker | cherry | 46 | 7 |
| wagon - break - radio | station | 51 | 14.57 |
| type - ghost - screen | writer | 54 | 9.37 |
| shine - beam - struck | moon | 62 | 6.17 |
| french - car - shoe | horn | 69 | 12.58 |
| hound - pressure - shot | blood | 72 | 6.98 |
| flake - mobile - cone | snow | 79 | 8.68 |
| water - mine - shaker | salt | 85 | 7.85 |
| cream - skate - water | ice | 90 | 4.12 |
| trip - house - goal | field | 13 | 8.02 |
| fence - card - master | post | 13 | 18.69 |
| dive - light - rocket | sky | 21 | 8.87 |
| spoon - cloth - card | table | 26 | 13.8 |
| tooth - potato - heart | sweet | 28 | 11.77 |
| rain - test - stomach | acid | 31 | 13.64 |
| tank - hill - secret | top | 38 | 11.2 |
| pile - market - room | stock | 44 | 7.42 |
| master - toss - finger | ring | 51 | 14.68 |
| wet - law - business | suit | 59 | 11.24 |
| pike - coat - signal | turn | 64 | 12.55 |
| aid - rubber - wagon | band | 69 | 6.51 |
| mouse - bear - sand | trap | 72 | 7.63 |
| date - alley - fold | blind | 85 | 7.06 |
| fountain - baking - pop | soda | 92 | 5.5 |
| *Note*. CRAs = Compound Remote Associates; Acc = Accuracy; RT = solution time; values = means. Normed CRAs including the reported Acc. and RT values were taken from [Bowden & Jung-Beeman, (2003)](https://www.zotero.org/google-docs/?wWFJxQ). | | | |

| **Table S2** | | | | | | |
| --- | --- | --- | --- | --- | --- | --- |
| Relationship between meta-cognitive prediction error and AHA dimensions *certainty* and *pleasure* | | | | | | |
|  | **Certainty** | | | **Pleasure** | | |
| Predictors | ß | CI | p | OR | CI | p |
| (Intercept) | 0.02 | -0.11 – 0.14 | <0.001 | 0.22 | 0.08 – 0.56 | 0.002 |
| RT | -0.25 | -0.29 – -0.20 | <0.001 | 0.96 | 0.95 – 0.98 | <0.001 |
| **PE_meta_** | **-0.03** | **-0.07 – 0.01** | **0.175** | **0.69** | **0.38 – 1.26** | **0.227** |
| accuracy | 0.57 | 0.53 – 0.62 | <0.001 | 24.05 | 14.57 – 39.68 | <0.001 |
| trial# | 0.01 | -0.03 – 0.05 | 0.639 | 1.00 | 0.99 – 1.01 | 0.366 |
| Random Effects | |  |  |  |  |  |
| σ^2^ | 2.05 |  |  | 3.29 |  |  |
| τ_00_ | 0.64 _subject_ |  |  | 4.87_subject_ |  |  |
|  | 0.05 _items_ |  |  | 0.12_items_ |  |  |
| ICC | 0.25 |  |  | 0.60 |  |  |
| Marg.R^2^ / Cond. R^2^ | 0.452 / 0.591 |  |  | 0.249 / 0.702 |  |  |
| *Note.* RT = solution time; OR = Odds Ratio; ICC = Intraclass Coefficient; Marg.=Marginal; Cond.=Conditional; PE_meta_ = meta-cognitive prediction error, RT = solution time.. | | | | | | |
|  |  |  |  |  |  |  |
|  |  |  |  |  |  |  |
| **Table S3** | | | | | | |
| Relationship between meta-cognitive prediction error and AHA dimensions *suddenness* and *surprise* | | | | | | |
|  | **Suddenness** | | |  | **Surprise** |  |
| Predictors | *ß* | *CI* | *p* | *ß* | *CI* | *p* |
| (Intercept) | 0.02 | -0.10 – 0.15 | <0.001 | -0.04 | -0.24 – 0.15 | 0.665 |
| RT | -0.34 | -0.39 – -0.30 | <0.001 | 0.22 | 0.17 – 0.26 | <0.001 |
| **PE_meta_** | **-0.06** | **-0.11 – -0.01** | **0.011** | **0.10** | **0.05 – 0.15** | **<0.001** |
| accuracy | 0.23 | 0.18 – 0.28 | <0.001 | -0.09 | -0.15 – -0.04 | 0.001 |
| trial# | 0.07 | 0.03 – 0.12 | 0.001 | 0.09 | 0.05 – 0.13 | <0.001 |
| **PE_meta_ × Acc.** |  |  |  | **0.04** | **-0.00 – 0.09** | **0.055** |
| Random Effects | |  |  |  |  |  |
| σ^2^ | 2.51 |  |  | 2.08 |  |  |
| τ_00_ | 0.52 _subject_ |  |  | 1.31 _subject_ |  |  |
|  | 0.00 _items_ |  |  | 0.02 _items_ |  |  |
| ICC |  |  |  | 0.39 |  |  |
| Marg.R^2^ / Cond. R^2^ | 0.277 / NA |  |  | 0.095 / 0.448 |  |  |
| *Note*. Acc = accuracy; RT = solution time; ICC = Intraclass Coefficient; Marg.=Marginal; Cond.=Conditional; PE_meta_ = meta-cognitive prediction error. We only report the best model fit. For suddenness, this is the model without PE_meta_*Acc. interaction term (but see Fig. S1, suddenness is predicted by a 3-way interaction with PE_meta_*Acc*RT). | | | | | | |


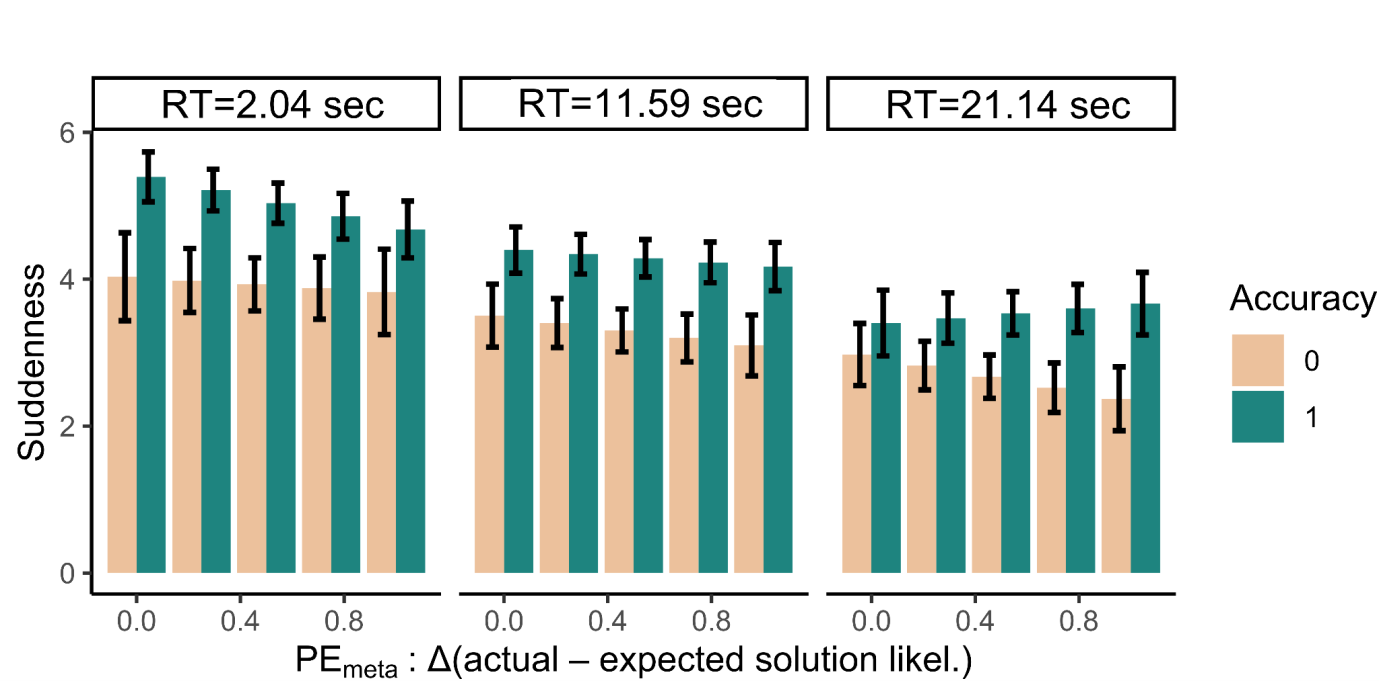


**Figure S1**. Relationship between suddenness and PE_meta_ mediated by solution time and accuracy

Note. RT = solution time; likel. = likelihood. Accuracy 0 = not correctly solved; Accuracy 1 = correctly solved. PE_meta_ = meta-cognitive prediction error. This figure depicts a three-way interaction between PE_meta_*RT*Accuracy to predict suddenness. To demonstrate this interaction, RT was split into three parts: lower, middle and upper RT (as is done automatically via the ggpredict function in R). The RTs in the boxes represent the respective means. The negative relationship between PE_meta_ and suddenness is mostly driven by fast solutions (~2.04sec).

***Control experiment: CRA task without prior rating task***

The theoretical and empirical support for the inclusion of the surprise component in the AHA experience has been well-documented (Gick & Lockhart, 1995; Danek et al., 2014, 2017; Webb et al., 2016). However, it is plausible that the rating task may have sensitized subjects to the surprise component in the CRA task, potentially biasing the results. To mitigate this bias and confirm the inclusion of the surprise dimension in the AHA experience, we conducted a repeat of the CRA task without the preceding rating task, using a new sample (n=48). The inclusion and exclusion criteria remained identical to those of the main experiment, resulting in the exclusion of six participants and a final sample size of n=42 [age (in years): *M*=47.7, *median*=49; *SD*=11.2; range = 27 – 70, 43.6% females]. Following the same procedure and analysis plan as in the main experiment, we estimated a measurement model for a latent AHA experience factor from all four dimensions (suddenness, pleasure, certainty, surprise) for correctly solved CRA items (RT>2sec). Consistent with the measurement model of the main experiment, we specified covariances between the pleasure and surprise variables (see Fig. S2).

The model converged normally after 29 iterations. The *Chi²* goodness-of-fit statistic (*Chi²*(1)=13.24, *p*<.001) was significant suggesting a significant difference between the measurement model and the data. However, practical fit indices suggested an acceptable fit of the model to the data (CFI= .926; RSME = .106; SRMR =.035). The latent insight factor loaded significantly positively onto *Certainty* (*λ*=.795, *z*=8.29; *p*<.001), *Suddenness* (*λ*=.321, *z*=6.00; *p*<.001) and *Pleasure* (*λ*=.307, *z*=3.95; *p*<.001) and significantly negatively onto *Surprise* (*λ*=-.291, *z*=-5.94; *p*<.001) suggesting that all four variables, including the surprise component, contribute significantly to the latent AHA experience factor (see Fig. S2). In sum, those results replicate the results from the main experiment and we have no reason to believe that the rating task may have biased the results.


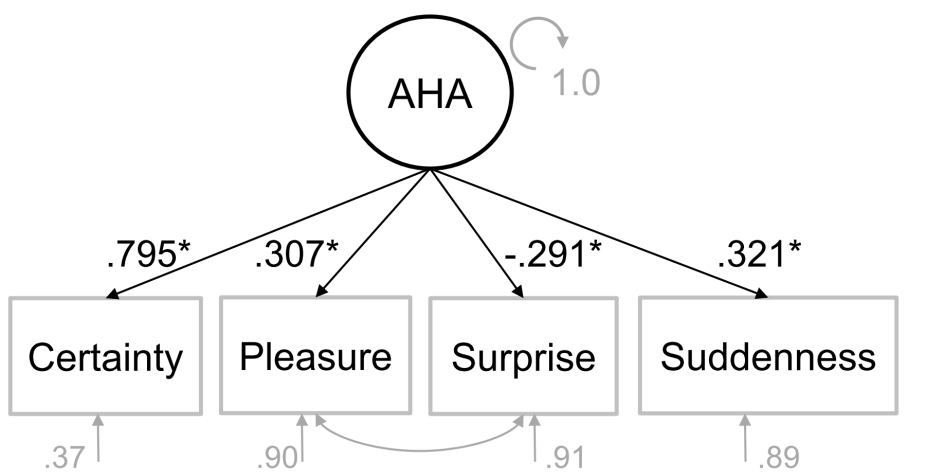


**Figure S2.** Control experiment: Latent AHA factor loading onto four different AHA dimensions

*Note*. Asterisk indicates significant factor loading at p<.001. Those results stem from a control experiment where subjects (n=42) executed the CRA task without prior rating task.
